# Glial fibrillary acidic protein: a potential biomarker for small fiber neuropathy?

**DOI:** 10.1007/s00592-025-02500-8

**Published:** 2025-04-07

**Authors:** Claus Vinter Bødker Hviid, Nicklas Højgaard-Hessellund Rasmussen, Johan Røikjer

**Affiliations:** 1 Department of Clinical Biochemistry, Aalborg University Hospital, Aalborg, Denmark; 2 Department of Clinical Medicine, Aalborg University, Aalborg, Denmark; 3 Steno Diabetes Center North Denmark, Aalborg University, Aalborg, Denmark; 4 Department of Endocrinology, Aalborg University Hospital, Aalborg, Denmark

**Keywords:** Diabetic polyneuropathy, Diabetes, Biomarkers, Glial fibrillary acidic protein, Quantitative sensory testing

## Abstract

**Background:**

Objective and easily applicable biomarkers for diabetic polyneuropathy (DPN) are warranted. Circulating nerve-specific proteins have emerged as valuable biomarkers for central nervous system disease but few of these have been tested in peripheral neuropathy. Glial Fibrillary Acidic Protein (GFAP) is highly expressed in non-myelinating Schwann cells while UCH-L1 is a neuron expressed stress protein not previous analyzed in DPN. In this pilot study, we explore serum GFAP and UCH-L1 levels in patients with/without DPN and controls.

**Methods:**

Persons with DPN (*n* = 28), without DPN (*n* = 31), and controls (*n* = 30) were evaluated in a cross-sectional design. Sural nerve conduction (velocity and amplitude) was evaluated by NC-stat DPNCheck™ and quantitative sensory testing of cold detection and pain was performed. GFAP and UCH-L1 levels were compared across study groups and the unadjusted correlation with nerve assessments evaluated.

**Results:**

Serum GFAP were lower in persons with DPN (20.9 ± 10.9 pg/ml) than in persons without DPN (26.2 ± 14.1 pg/ml) (*p* = 0.04) or controls (31.7 ± 26.0 pg/ml) (*p* = 0.02). GFAP levels were not different in persons without DPN and controls (*p* = 0.61). UCH-L1 levels were not different between study groups (*p* = 0.48). GFAP levels correlated with cold pain threshold (Rho= − 0.320, *p* = 0.02) but failed to reach significance for cold detection (Rho= − 0.236, *p* = 0.09). No correlation was observed between GFAP and nerve amplitude (*p* = 0.58) or conductivity (*p* = 0.86).

**Conclusion:**

Serum GFAP levels are reduced in persons with DPN compared to persons without DPN and controls. Reduced serum GFAP levels may be associated with reduced markers of small nerve fiber damage obtained from quantitative sensory testing in people with diabetes.

## Background

Diabetic polyneuropathy (DPN) is a common and debilitating complication of diabetes and associated with substantial health care costs [1]. Up to 50% of all people with diabetes suffer from DPN and half of these develop neuropathic pain [2, 3]. This is associated with considerably morbidity, reduced quality of life, increased mortality, and substantial health care costs [1]. Despite this, progress in development of DPN treatment has lacked behind [4]. This may, partially, stem from the lack of sensitive and objective tools to detect and monitor DPN in its earliest stages and evaluate potential effects in treatment trials.

The most common form of DPN is a length-dependent symmetrical polyneuropathy that preferentially target small peripheral nerves while the affection of larger myelinated fibers is usually a late phenomenon [5]. The pathophysiology behind these changes remains incompletely understood but metabolic derangements and inflammatory activation are key factors driving the cellular stress leading to Schwannopathy and axonopathy [6, 7].

The last decade has seen a rapid progression in development of blood-based biomarkers of acute- and degenerative central nervous system (CNS) diseases [8, 9]. Using ultra-sensitive technologies, nerve-specific proteins can be measured in peripheral blood and provide easily assessable tools to diagnose, monitor and prognosticate diseases acute injuries and degenerative diseases of the CNS [8, 9]. Such biomarkers are currently being integrated in clinical care of patients with CNS diseases and have been crucial in development of novel disease-modifying drugs [10–12]. However, the potential of blood-borne nerve-cell derived proteins as biomarkers for diseases of theperipheral nervous system has received little attention [13].

The structural neuroaxonal protein, neurofilament light chain (NfL) have proven potential as a blood-borne biomarker for DPN [14–16]. NfL is a cytoskeletal protein with neuron-restricted expression that is predominantly expressed in large-caliper, myelinated axons [8]. It is released during neuron decay and would expectably be most optimal for detection of large-fiber neuropathy. Other candidates among nerve-restricted proteins may therefore hold potential to reflect the involvement of other cell-types or aspects of the pathophysiology but this remains largely unexplored.

Glial Fibrillary Acidic Protein (GFAP) is a cytoskeletal protein expressed in astrocytes, non-myelinating Schwann cells and enteric glia [17]. The protein plays a critical role in gliosis and is highly upregulated in acute and degenerative CNS-diseases [9, 18]. Blood-levels of GFAP have been intensively investigated as biomarker for CNS glial reaction and is approved for clinical biomarker use [8, 12, 19]. Ubiquitin C-terminal hydrolase-L1 (UCH-L1) is a highly abundant, neuron-restricted cytoplasmic protein expressed in central and peripheral neurons [20]. It has a key role in regulation of the unfolded protein response, a cellular process that removes toxic misfolded and oxidized proteins that accumulate during cellular stress [21]. The expression of UCH-L1 is an absolute requirement for maintenance of axonal integrity and blood-levels of UCH-L1 are also approved for clinical biomarker use [12, 19]. As such, blood-levels of GFAP and UCH-L1 may reflect aspects of DPN pathophysiology but their biomarker potential in DPN remains unexplored.

We performed an exploratory study to evaluate blood-levels of GFAP and UCH-L1 in diabetics with and without DPN. In a cross-sectional design, GFAP and UCH-L1 were measured in 28 persons with diabetes and DPN (DPN+), 31 persons with diabetes without DPN (DPN-) and 30 age- and sex-matched controls. Outcomes were GFAP/UCH-L1 levels in the study groups and their correlation with measures of large- and small-nerve fiber nerve affection.

## Materials and methods

### Study cohort

The present study was conducted using biobank material form the DIAFALL cohort [22] which was conducted at Steno Diabetes Center North Denmark, Aalborg University Hospital, from April 2019 until June 2021. Participants for the present study were collected from a sub-cohort (*n* = 103), designed to assess diabetic neuropathy that was included between April 2022 to September 2022 [23]. The trial was conducted in accordance with the declaration of Helsinki, all applicable laws and regulations and complied with Harmonized Tripartite Guideline for Good Clinical Practice. It was approved by the regional ethical committee (No: N-2019-0004) and was registered at Clinicaltrials.gov (NCT05389566).

The DIAFALL trial is a single center, cross-sectional, clinical trial which includes people with type I Diabetes (T1D) and type 2 Diabetes (T2D) as well as healthy controls [22]. Participants with T1D and T2D duration of more than a year and being between 40 and 80 years of age were included. Control persons were included if between 20 and 80 years and not having diabetes. Participants were excluded if suffering from Maturity-onset Diabetes of the young; having moderate to severe liver (ALT > 250 u/L) or kidney dysfunction (eGFR < 15mmol/L/1.73m2); if being pregnant or breast feeding; suffering from active malignancy or being terminally ill; having current or recent (within one year) alcohol or drug abuse; or unable to communicate in Danish. Participation in other trials or performing more than 10 h exercise/week were also reasons for exclusion. Lastly, participants judged by the investigator as unable due to lack of understanding or physical ability were excluded.

For the present study, a sub-cohort of age- and sex-matched people with T1D and T2D with diabetic polyneuropathy (DPN+) or without diabetic polyneuropathy (DPN-) along with controls without diabetes was extracted from the cohort. People with diabetes were classified as DPN + based on the presence of a combination of symptoms and signs, which included any two or more of the following: neuropathic symptoms, decreased distal sensation, or unequivocally decreased or absent ankle reflexes (Toronto Consensus “Probably neuropathy”) [5].

### Data collection

In the DIAFALL study participants were subject to extensive investigation. All clinical examination procedures were validated through monitoring of Coefficient of Variance (CV) for each procedure. Only, CV below 10% were considered sufficient.

All participants completed a standardized questionary on anamnestic information that included diabetes status, diabetes complications, osteoporosis, smoking status, alcohol consumption and use of medications.

Body mass index (BMI) was calculated as weight in kilograms (nearest 0.1 kg) divided by height in meters (nearest 0.5 cm) squared. Weight was measured using a column scale and height using a stadiometer (both from: Seca Gmbh & co, Hamburg, Germany).

### Nerve conduction tests

#### Large fiber assessment

The function of large nerve fibers was evaluated using bedside nerve conduction studies device NC-stat DPNCheck™ (NeuroMetrix, Walthan, USA). Conduction velocity and amplitude of the right sural nerve was investigated. The Vibration Perception Threshold was evaluated by biothensiometry (Biomedical Instrument CO, Newbury Ohio, USA) on the proximal aspect of first toe on each foot. The amplitude slowly increased until vibration was registered, then reduced until no longer detectable. The test was repeated twice, and the CV was 2.3%.

#### Small fiber assessment

Small fiber assessment was done by thermal quantitative sensory testing (QST) on the dorsum of the right foot using a TSA-2001 Neurosensory Analyzer (Medoc, Ramar, Yishai, Israel). The temperature was changed at a rate of 1 °C/sec. and the participant indicated the threshold. Measurements were performed in the order of: Heat detection, cold detection, heat pain detection, cold pain detection. Each variable was assessed three times consecutively and the CV was 9.9%.

### Blood sampling and routine biochemistry

Blood samples were collected by standard operating procedure by certified research laboratory technicians from Steno Diabetes Center North Denmark, Aalborg University Hospital. Samples were analyzed in our accredited laboratory (ISO/DA18959) using methods validated and optimized for routine clinical use.

### Laboratory analysis

UCH-L1 and GFAP were measured in serum samples on an Alinity-I module using the TBI assay by Abbott (Abbott, Abbott Park; Illinois, USA). The TBI assay is an automated panel analysis that uses proprietary reagents and measures GFAP and UCH-L1 by chemiluminescence technology. The assay is established in our accredited laboratory (ISO/DA18959). The assay as a LoD of 3.2 ng/L (GFAP) and 18.3 ng/L (UCH-L1) and the linearity range from 6.1 to 42.000 ng/L (GFAP) and 26.3 to 25.000 ng/L (UCH-L1). In our hands, the imprecisions for GFAP are 3.1% (level 24.1ng/L), 2.5% (level 485.3 ng/L) and 2.1% (level 30,521.4 ng/L). The imprecisions for UCH-L1 are: 2.0% (level 258.9ng/L), 1.8% (level 2042.1ng/L), 1.9% (level 15,133.0 ng/L). The reference intervals for this assay are 6.6–70.9 ng/L (GFAP) and 44.7 to 226.8 ng/L (UCH-L1).

For this study, serum samples were thawed at room temperature and centrifugated (2000 g, 5 min, RT) to remove any debris. Samples were run in singles by two certified laboratory technicians experienced with the assay and blinded to the study. Per routine samples below the LoD is rerun and samples above the upper linearity limited are diluted and rerun. In this study all samples were with the quantification range, and none were rerun. Samples were batch analyzed over two days using a single reagent LOT.

### Statistics

The data distribution was evaluated by visual inspection of inverse QQ-plots. Data are presented as absolute numbers with percentages, means with standard deviation (SD) or 95% confidence intervals (CI), or medians with interquartile ranges (IQR) depending on the data distribution. Group-wise comparison was done by a one-way analysis of variance (ANOVA) or Kruskal-Wallis test as appropriate. Comparison of two groups was done by an unpaired students t-test or Mann-Whitney U-test as appropriate. The biomarker levels were positively screwed and therefore ln-transformed before the statistical comparison. Associations between biomarkers and nerve conduction tests (conductivity and amplitude) aswell as cold-detection and cold-pain threshold were analyzed in all persons with diabetes by Spearman’s Rho.

All analyses were performed in STATA 18.0 and a p-value of 0.05 was considered statistically significant.

## Results

General participant characteristics are presented in Table 1. The fraction of T1D or diabetes duration did not differ between the DPN + and DPN- groups but DPN + had slightly higher HbA1c than those with DPN-. The BMI, burden of cardiovascular- and kidney disease was not different between the groups. People with + DPN had reduced nerve conduction velocity and amplitude compared with those with -DPN. A tendency to increased cold-QST was observed among DPN + compared with DPN- but this did not reach significance.

**Table 1:** Cohort charateristics

	Diabetes + DPN (*n* = 28)	Diabetes– DPN (*n* = 31)	Healthy controls (*n* = 30)	*p*-value
Age, years	65.0 ± 6.9	64.8 ± 6.7	65.5 ± 5.0	ns
Sex, % male	71%	71%	70%	ns
Body Mass Index	27.5 ± 4.2	26.5 ± 4.4	27.2 ± 4.5	ns
Diabetes type, % type 1	39%	39%	N/A	ns
Diabetes duration, years	17 (7;36)	17 (10;26)	N/A	ns
Haemoglobin A1c	66 (58;70)	58 (49;67)	37 (35;37)	ns*
Nephropathy, %	3.6%	6.5%	3.3%	ns
Atrial fibrillation, %	7.1%	0.0%	0.0%	ns
Previous acute myocardial infarction, %	3.6%	9.7%	0.0%	ns
Ischemic heart disease, %	3.6%	16.1%	3.3%	ns
Previous heart failure, %	3.6%	0.0%	0.0%	ns
Previous apoplexy/TCI. %	3.6%	6.5%	0.0%	ns
Sural nerve conduction velocity, m/s	35.0 (0.4;49.0)	45.5 (35.0;50.0)	–	*p* < 0.01
Sural nerve amplitude, µV	4 (2;5)	7 (5;9)	–	*p* < 0.05
Cold Detection Threshold, ^◦^ Celsius	27.8 (23.8;28.0)	24.4 (19.1;27.6)	–	ns
Cold Pain Threshold, ^◦^ Celsius	2.2 ± 4.5	5.0 ± 8.6	–	ns

Data presented as means ± sd when normally distributed or as medians with interquartile ranges when non-normally distributed

Differences between groups are calculated using ANOVA or Kruskal–Wallis H test depending on distribution

Nephropathy defined as EGFR < 60 mL/min/1.73 m^*2*^

* Difference between DPN+ & DPN−

**Fig. 1:**
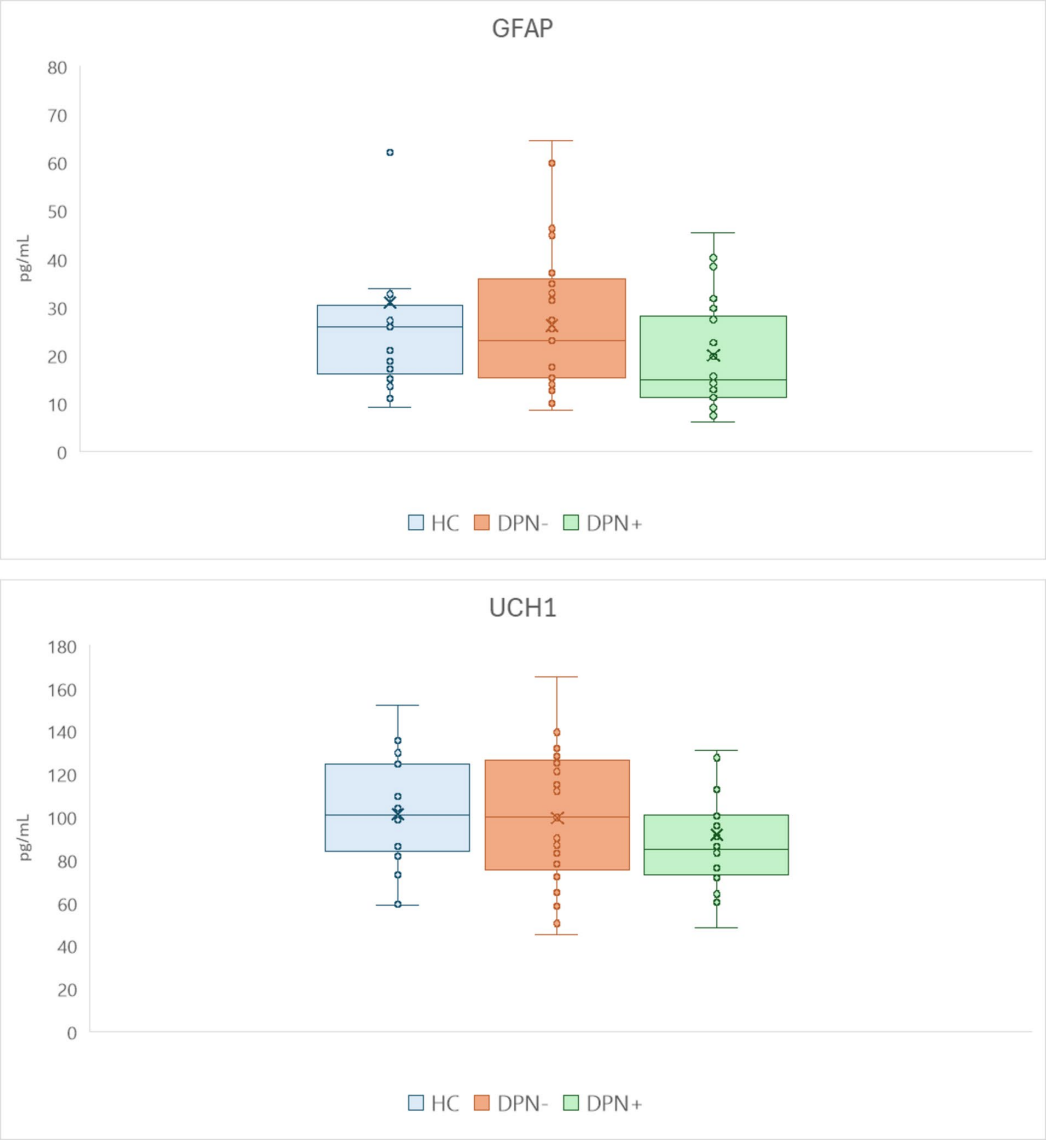
Serum GFAP and UCH-L1 levels in controls (HC, *n* = 30) and persons without (DPN-, *n* = 31) and with (DPN+, *n* = 28) diabetic polyneuropathy. Levels of GFAP in controls, DPN− and DPN + were 31.7 ± 28.0, 26.2 ± 14.1, and 20.0 ± 10.9 pg/ml, respectively. Levels of UCH-L1 in controls, DPN-, and DPN + were: 91.3 ± 25.7, 99.6 ± 30.5, and 91.7 ± 34.3 pg/ml, respectively. Boxes indicate median and interquartile range. Whiskers are adjacent values.

The GFAP and UCH-L1 measurements are presented in Fig. 1. Serum GFAP levels were significantly reduced in people with DPN + compared with DPN- and healthy controls. Levels were not different between DPN- and health controls. No difference was observed between UCH-L1 levels between the study groups.

GFAP levels correlated with cold pain threshold (Rho = − 0.320,*p* = 0.02) while the correlation with cold detection threshold failed to reach significance (Rho = − 0.236, *p* = 0.09). No correlation was observed between GFAP levels with sural nerve amplitude (rho = − 0.078, *p* = 0.58) or conductivity (Rho = 0.025, *p* = 0.86).

## Discussion

This present study provides the first evidence for altered serum GFAP levels in persons with DPN. It provides indications, also for a correlation between serum GFAP levels and small, but not large, nerve fiber affection in people with diabetes. Collectively, this is suggestive of a previously un-appreciated potential of circulating GFAP as biomarker for early-stage DPN.

The pathophysiology behind DPN is incompletely understood but metabolic derangements in an inflammatory environment are key elements that drive cellular stress leading to Schwannopathy and axonopathy [6, 7]. Schwann cells are the most abundant cell-type in the peripheral nerve system and include myelinating and non-myelinating types [6]. Schwann cells are indispensable for maintenance of neuroaxonal integrity and functioning and impediment of their normal function by the metabolic imbalances of diabetes holds a prime role in the pathophysiology of DPN [6]. As GFAP is a cytoskeletal protein, expressed in the non-myelinating Schwann cells of the peripheral nervous system, it is biologically plausible for this protein to be regulated in persons with DPN [18]. To our knowledge, circulating GFAP levels has been investigated in few prior studies on peripheral neuropathy [24–28]. Increased circulating GFAP was observed in inflammatory and chemotherapy-induced neuropathies [25–28]. While this is at odds with our findings, the pathophysiological underpinnings of DPN are clearly different from those of inflammation and pharmacologic toxicity. As such, the converse GFAP regulation in a different pathophysiological setting is plausible. Only one prior study investigated circulating GFAP levels in persons with type 2 diabetes but used an assay unable to detect serum GFAP in both patients and controls [24]. However, this study also investigated serum S100 calcium-binding protein B (S100b) and reported reduced levels in persons with DPN [24]. S100b is a member of the calcium-binding s100 protein family which is highly expressed in glia (including Schwann cells) and as such, these findings support our data [18, 24]. While S100b reductions in DPN is not uniformly reported [29] a recent high quality, prospective study, designed to identify biomarkers of DPN uncovered additional supporting evidence [30]. This study demonstrated reduced expression of the Schwann cell specific protein, Myelin Protein Zero (MPZ), in Schwann cell cultures grown under metabolically stressful conditions and showed reduced levels of MPZ in nerve biopsies from persons with DPN. Lastly, that circulating levels of MPZ mRNA were reduced in persons with T2D and DPN, discriminated DPN + from DPN- with high accuracy, and correlated with neuropathy scores as well as small- and large nerve fiber affection. Altogether, these lines of evidence appear in favor of our observation of reduced serum GFAP in persons with diabetes complicated by DPN. We investigated also serum levels of the neuron stress protein, UCH-L1 to capture the cellular stress that eventually manifests as peripheral nerve decay. As prior studies have uncovered a biomarker potential of circulating neurofilaments [14, 15, 30], measurements of UCH-L1 would theoretically represent earlier phases of DPN development. Whether UCH-L1 was not regulated in our study due to its design, study population or inappropriateness of the biomarker remains unknown. Collectively, our data encourage further investigations in larger, prospective cohorts to unravel the biomarker potential of GFAP in DPN.

The persons in our cohort had a relatively short diabetes duration and were categorized with probably neuropathy according to the Toronto Consensus Criteria [5]. In line with this, the nerve conduction studies suggested a mild neuropathy burden only. In these mildly affected persons, serum GFAP levels correlated with quantitative sensory testing results which imply the biomarker to reflect early pathological events during DPN development. It is biologically plausible that non-myelinating Schwann cells in the Remark bundle are involved in early stages of DPN, commonly characterized by symptoms of small nerve fiber affection [6]. This calls for further studies to investigate longitudinal changes in serum GFAP with DPN progression. Such investigations should include comparative evaluation of GFAP and NfL to uncover the time-differential differences in neuron and glia biomarker affection and the potential benefit of combining such biomarkers.

From a biomarker perspective, the large overlap between study groups could be a concern in terms of diagnostic capabilities. Due to our limited sample size, we did not attempt to evaluate the diagnostic performance of serum GFAP. We have previous reported a relatively large inter-individual and a small intra-individual variation of serum GFAP [31] which correspond with the current data. With these characteristics, GFAP would mainly be a biomarker for monitoring and prediction of DPN– akin NfL [14, 15, 32]. Another technical consideration is on the choice of biochemical GFAP assay. Several GFAP isoforms exist, are expressed in a tissue-specific manner, and subject to post-translational modifications [18]. In this study, we used a proprietary assay with no information available on the target epitopes. Different assays would be expected to capture GFAP-isoforms selectively which warrants caution in choice of methodology and comparison of results across studies. If GFAP is cooperated as a biomarker for DPN in follow-up studies, work is required to define the GFAP isoform that reflects the pathophysiological process of DPN development most accurately.

Our study has a number of limitations to consider. First, this is a small, exploratory study with a cross-sectional design and should be interpreted accordingly. However, even in this setting and with considerable variation in the biomarkers investigated, a clear reduction in GFAP was observed which suggests a true effect. We matched the study groups on age which is well-described and strong confounding factor for serum GFAP levels [9, 33]. The distribution of other relevant covariates with a known effect on serum levels of neurological proteins, such as BMI, cardiovascular, kidney or hepatic disease [34], were evenly distributed between the groups. They are therefore considered not to have impacted our results critically. However, the effect of unknown confounders cannot be excluded. We included persons with T1D as well as T2D in our cohort. Differences in pathophysiological mechanisms may exist between diabetes types which could not be addressed with the sample size available in this study. Future studies should address this. Being a glial protein, the bulk expression of GFAP is in the CNS [18] which is of major importance in interpretation of our results and for potential clinical applicability. Persons with T1D demonstrate signs of cerebral degeneration, and more so if suffering from DPN [35–37]. We did not evaluate whether this occurred in our cohort. However, prior studies on serum GFAP in persons with neurodegenerative diseases uniformly reported elevated levels [9]. As such, the presence of neurodegeneration would have further strengthened our results. The clinical applicability of serum GFAP in a population with high incidence of vascular disease warrants caution as GFAP is a sensitive biomarker for (sub)-acute injuries to the CNS [9]. This is a fundamental obstacle to the use of any nerve protein, also expressed in the CNS, as biomarker for neuropathy. Consequently, results would have to be interpreted in their clinical context as for any biomarker in clinical use.

## Conclusion

Serum GFAP levels are reduced in persons with DPN compared to persons without DPN and to controls. Reduced serum GFAP levels may be associated with reduced markers of small nerve fiber damage obtained from quantitative sensory testing in people with diabetes.
